# Application of Augmented Reality Technology in Children’s Picture Books Based on Educational Psychology

**DOI:** 10.3389/fpsyg.2022.782958

**Published:** 2022-02-03

**Authors:** Rui Wang

**Affiliations:** College of Art and Design, Beijing Polytechnic, Beijing, China

**Keywords:** picture books for children, mental cognition, the image of the mythical beast, interview method, the behavioral intention

## Abstract

To cultivate children’s imagination, observation, thinking ability, and aesthetic consciousness, the questionnaire survey is adopted to analyze the design strategies and principles of children’s picture books based on augmented reality (AR). Primarily, the related concepts and theories are expounded for the research content. Children in preschool aged 4–5 years are invited as primary participants in this work, and the psychological characteristics of the invited children are analyzed in depth. Then, a study is carried out on the existing AR children’s picture books. The problems existing in the design of AR children’s picture books are found, and then, related solutions are put forward based on the results of the questionnaire survey. Besides, a design is made on the strategies and interactive design principles of AR children’s picture books on mobile terminals that are more in line with the needs of children. The results show that 41.07% of parents do not understand AR technology, and 37.5% of preschool children indicate that they do not operate mobile devices independently. However, they need the assistance of parents to use this kind of picture book. A total of 44.64% of parents believe that the main problem of AR picture books in the current market is the lack of interesting interaction. Given the above problems, five principles are proposed for the design of AR children’s picture books based on mobile terminals, namely, easy operation principle, interesting principle, guiding principle, timely feedback principle, and safety principle. A set of universally applicable design methods are proposed for AR children’s picture books based on mobile terminals, which provides certain theoretical guidance for the development of related types of products.

## Introduction

The development of visual culture has caused a great change in people’s reading styles. Children are in a critical period of physical and psychological development. Picture books have gradually developed into an essential tool, playing an important role in children’s cognition development (Patterson, 2019; Fears and Lockman, 2020). During the reading process, picture books can help children to establish the correct three outlooks. As a type of literary carrier, picture books have brought a new era of text expression and image portrayal, which helps to enhance children’s interest in reading. The picture book is a special educational way for children, which can satisfy children’s imagination and desire for exploration and play a significant role in children’s growth (Takacs and Bus, 2018; Yamaji et al., 2020). Precisely, picture books have become the primary choice for parents and schools to cultivate and educate their children because of these characteristics. In China, translations imported from other countries account for the majority of the market of picture books for children. In contrast, the market share of Chinese picture books for children is limited. According to the development law of children’s mental cognition, picture books should actively absorb high-quality resources worldwide and develop, following the actual situations.

Regarding the relationship between picture books for children and child psychology development, Sundmark (2018) described the development trajectory of early concept books as picture books for children. Based on the analysis of language and visual dimensions, the results revealed the importance of picture books in the cognitive development of children and adolescents (Sundmark, 2018). Candel et al. (2020) reckoned that picture books provided non-linguistic subjects, including art, numbers, and music, from where children could study knowledge, culture, and communication, and improve their ability of cognition. Scholars recommended using Cuadernia software to compile picture books and emphasized their importance in broadening students’ knowledge and enhancing students’ learning vitality and interest (Candel et al., 2020). To help children obtain beneficial information from emotional cognition and emotional preference, Wang (2017) used different artistic styles, emotional themes, and color elements to design picture books and explore children’s emotional states and emotional expressions. Aldrich and Brooks (2017) chose a picture book about frogs to study the relationship between children’s narrative evaluation and performance. At present, experts and scholars have researched the relationship between picture books and the cognitive development of children. Whether in China or other developing countries, products of children’s picture books with augmented reality (AR) are not very mature, basically in the stage of development and exploration. The product design is still relatively immature. There are many products put into the market, but due to the lack of targeted research literature, there is no corresponding reference in the research process, especially in the application design of AR children’s picture books in the mobile terminal. Related researches focus on the realization and improvement of AR technology and do not focus on the content of products. There is a lack of further research on how to use AR technology to provide more benefits to children’s picture books (Billinghurst et al., 2001).

Based on this, the research and design are mainly focused on the AR children’s picture book in mobile APP. Preschool children aged 4–5 years are invited as the participants of the research. Entertainment elements are added in AR children’s picture books concurrently with these picture books providing education and learning for children’s users, to promote children’s intellectual development, improve children’s aesthetic level, and make children and their parents get different reading experiences.

## Literacy Review

### Deep Study Into This Research

From the perspective of academic attention and academic communication, taking 2015 as the dividing line, this work divides the domestic research on deep learning into two stages: from about 2004 to 2015, deep learning entered the vision of educational researchers in China, which is the dormant period of deep learning; since 2015, deep learning has developed rapidly in China. Overall, the domestic research on deep learning has not yet formed systematic. Since 2004, deep learning has not caused a boom like other nouns in China. On the contrary, it is in a dormant state. Only a few researchers have introduced it at the theoretical level. It may be that the theory of deep learning itself remains to be improved, followed by deep learning. It is almost a reversal of traditional teaching, and the goal level is far from the general goal of school education and teaching. Finally, the conditions to promote and support deep learning are not mature or even lacking, such as the creation of real learning situations, and complex abstract content is difficult to visualize. With the application of emerging technologies in education and teaching gradually opening up, deep learning has entered a period of rapid development after a long dormant period. Since the beginning of 2015, both the number of papers published by deep learning and the degree of academic communication and attention have continued to rise. Specifically, from 2015 to the first half of 2016, deep learning has entered a peak period of development and has maintained a small growth since then. Researches are concentrated in the universities of the Chinese Academy of Sciences, Wuhan University and Tsinghua University, covering automation technology, computer software and computer applications, secondary education, and primary and higher education; the scope involved includes artificial intelligence, machine learning, and neural network. This section will focus on the research status of deep learning in education and teaching.

The researches on deep learning in developed countries dominated by the United States mainly focus on two major issues, namely, how to promote the occurrence of deep learning and how to evaluate deep learning, including the evaluation methods of deep learning effect and the evaluation methods of promoting the occurrence of deep learning. Litjens et al. (2017) reviewed the main concepts of deep learning related to image analysis, summarized more than 300 contributions to this field, investigated the application of deep learning in image classification, object detection, segmentation, registration, and other tasks, and provided a brief overview of research in each application field. Chen et al. (2017) introduced the concept of deep learning into hyperspectral data classification. The applicability of a stacked automatic encoder is verified according to the classical classification method based on spectral information, and a classification method based on spatial dominance information is proposed. Then, a new deep learning framework is proposed to fuse these two features, to obtain the highest classification accuracy. Oshea and Hoydis (2017) proposed and discussed several new applications of physical layer deep learning. By interpreting the communication system as an automatic encoder, a basic new method is developed to treat the communication system design as an end-to-end reconstruction task, aiming to jointly optimize the transmitter and receiver components in a single process.

### Present Researches on Children Picture Books

As the origin of picture books, Europe and the United States have gradually matured after hundreds of years of growth and formed a relatively complete cultural industry. The increase in the incentive systems such as the International Andersen Awards and the Caddick Awards has made the institutional system of picture books more and more perfect, which to some extent represents the high level of the picture book market. As early as 1,744, Mr. Newbury’s *Beautiful Book* was published. It was the first book specially designed for children in the world, creating a precedent for foreign picture books and gaining the title of “father of children literature.” Foreign studies on the content of picture books are relatively more in-depth, such as Perry Norman’s *Saying Pictures: Narrative Art of Children’s Picture Books* mainly explored the important relationship between pictures and texts, using pedagogy, psychology, aesthetics, and other arguments to clarify the overall significance of children’s picture books.

Jennifer (2019) proposed an art project to strengthen the scientific content of ecology in children’s picture books. Ten students from different socioeconomic classes and different cultural and linguistic backgrounds from the United States and Spain participated in the event. Next-generation scientific standards have been identified and resolved in these art projects. Students’ understanding and participation are assessed through teacher’s observation, photographs, and student attitude surveys. Strouse and Ganea (2021) pointed out that story picture books with examples can be used to teach children the scientific concepts. Learners can abstract relational information by comparing examples in the book, to have a more abstract and transferable understanding of the concept. Breit-Smith et al. (2017) discussed that the effect of interactive book reading intervention is characterized by illustrated picture books. The group intervention was conducted by four practitioners three times a week for 8 weeks, targeting six preschool children identified as having language disorders. Interventions include the use of language facilitation strategies in interactive book reading and postreading extension activities to promote children’s understanding of sequential text structures, academic vocabulary, and plant-related scientific subject knowledge.

## Materials and Methods

### Theoretical Basis

**FIGURE 1:**
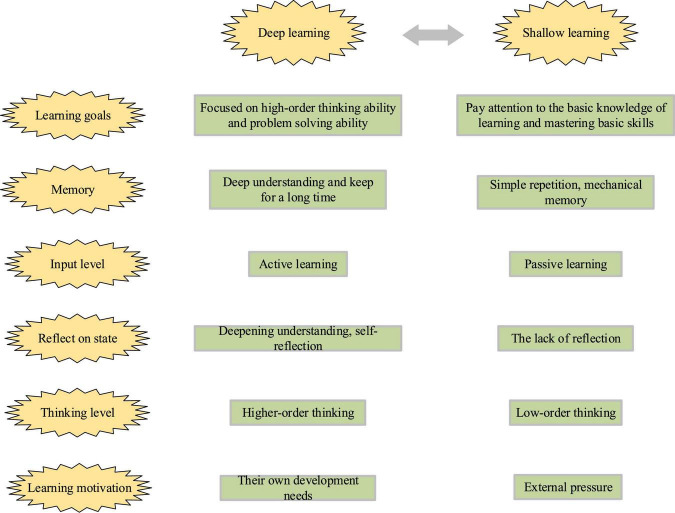
Comparison of deep learning and shallow learning.

(1) Concepts and Features of the Deep Learning
The new era puts forward higher requirements for talent cultivation. Countries around the world are constantly exploring how to cultivate talents that can adapt to the needs of future social development. Based on this background, the term of core literacy required for future life comes into being, and the proposal of deep learning is an active response to talent cultivation in the new era. Deep learning is proposed based on the widespread phenomenon of learners’ non-participation, one-way transmission of knowledge, learners’ passive mechanical acceptance, and the adverse consequences of shallow learning. Based on the investigation of the current situation of children’s picture book teaching, it is not difficult to find that the current children’s picture book lacks scientific activities of application, and children are still in a passive and mechanical learning state. It is far from the training objectives of the curriculum. Therefore, deep learning has become a learning method that matches the training objectives of children’s teaching courses. Meanwhile, it has become one of the effective ways to change the participation status of learners and shallow learning.
Deep learning is a concept proposed by American scholars in the second half of the 20th century. Its initial purpose is to explore the degree of learners’ learning engagement and mastery of knowledge. In the learning process, different learners will adopt different strategies to achieve the purpose of mastering knowledge. Learning methods can be simply divided into deep learning and shallow learning. Deep learning refers to learners’ thinking, understanding, and raising their questions in the learning process; shallow learners do not pay attention to the understanding of knowledge, only through passive memory to obtain knowledge. Deep learning is superior to shallow learning. Further deep learning and shallow learning are compared, as shown in Figure 1.

**FIGURE 2:**
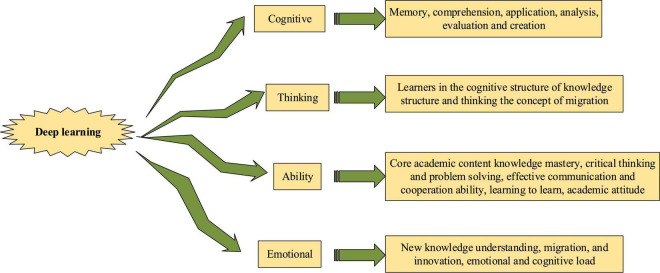
Different definitions of deep learning.

In the process of continuous development, the concept of deep learning is also defined by educational researchers at home and abroad from different perspectives, as shown in Figure 2.

**FIGURE 3:**
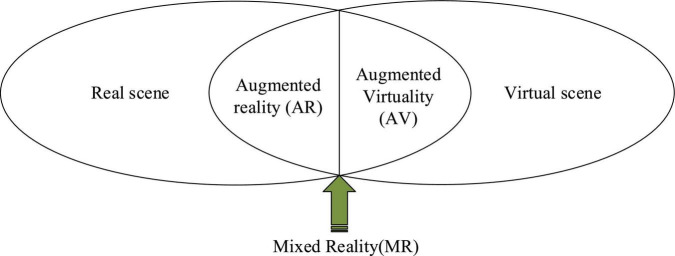
The relationship between AR, MR, and AV.

Through the interpretation of the concept of deep learning from the above different perspectives, it is found that to promote the occurrence of deep learning, school teaching must fully understand its connotation and characteristics, design corresponding learning activities, and guide learners to conduct deep learning. This paper elaborates on the characteristics of deep learning from the perspective of learners.
Learners are the main participants in learning activities and the actual executor of learning tasks, especially in deep learning. The prevalence of shallow learning is closely related to learners’ passive, negative learning attitude, self-rule orientation, and vague learning objectives. Depth has the following characteristics: learners try to understand the learning content in-depth and have critical learning of the knowledge content presented by teachers; actively seek the connection between old and new knowledge, build a new cognitive structure; explore the reasons and laws behind the conclusion, logical reflection, whereas shallow learning is manifested as passive mechanical acceptance of knowledge information; not actively seeking the internal relationship between the old and new knowledge points; simple acceptance of the conclusions, lack of inquiry process, biased toward the conceptual facts of the mechanical memory; learning aims at coping with exams. Learners’ active participation is the key to deep learning, which is reflected in the comprehensive input of perception, thinking, emotion, will, and values; promoting the comprehensive development of specific people is the basic way to cultivate learners’ core literacy.
(2) Augmented Reality Technology
Augmented reality technology is not a new concept, belongs to the field of virtual reality research, and is an important direction of human–machine interface interaction technology development. In the early 1990s, Tom Caudell and his colleagues first proposed the term “AR” in the design of auxiliary wiring systems. They superimposed the wiring path drawn by simple lines and text prompt information in real time, to help the mechanic complete the whole disassembly process quickly and efficiently. Subsequently, AR applications have emerged in medical, military, entertainment, manufacturing, and maintenance fields. There are two widely accepted definitions of AR. The “reality-virtual continuum” was initially proposed by Milgram and Kishino. This definition classifies the environment presented by the combination of virtual objects and real scenes. It is considered that the real scene and the virtual environment are located at both ends. The part near the real environment is AR, and the part near the virtual environment is augmented virtuality (AV). The middle part is mixed reality (MR), as shown in Figure 3.

**FIGURE 4:**
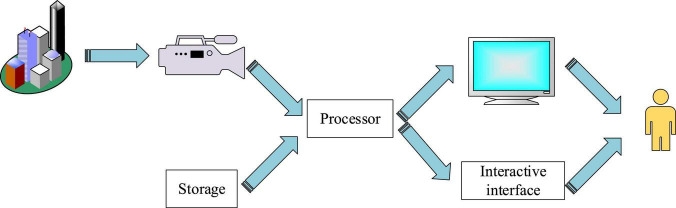
Systematic structure of AR technology.

The second definition was proposed by Ronald Azuma of the University of Bekaa in Azuma, arguing that AR technology has three basic characteristics, namely virtual–real combination, three-dimensional registration, and real-time interaction. The first definition shows that AR technology is more inclined to the real world, and the virtual object is constructed in the computer and superimposed into the real scene by graphic image technology. The three-dimensional registration feature is reflected in AR that the position information of the camera is tracked in real time to determine the accurate position of the virtual object in the real scene and realize the complete fusion of the virtual and real scene. The real-time interactive feature is that users can get feedback information in time. In summary, AR technology is the extension and development of virtual reality technology. This technology provides users with a virtual and real combination environment by simulating objects, which can see the generated virtual objects. More importantly, users can manipulate and control virtual model objects, such as rotation. AR intention realizes the superposition of computer-generated objects and real situations, that is, the effect of combining virtual and real. Using a variety of devices, such as eyes with imaging primitives, overlaying virtual objects into real scenes allows users to see virtual objects and real scenes at the same time and achieve interaction with virtual objects. AR research involves a wide range, including computer graphics and image processing, human–machine interface interaction, and psychology. Although it does not need to display the complete scene, it needs to analyze much positioning data and scene information, to ensure that the generated virtual objects can be accurately located in the real scene. Therefore, an AR system includes four basic steps: first, to obtain the relevant information of the real scene; second, the real scene and camera position information are comprehensively analyzed and then generate virtual scene; finally, it is displayed directly or combined with the real scene video. The complete AR system components include camera unit, storage unit, processor unit, display unit, and human–computer interaction interface unit, as shown in Figure 4.

The camera unit is used to shoot the real scene; the memory unit is divided into local storage and cloud storage; as the core unit of the whole system, the processor determines whether the whole system can operate efficiently. The display unit mainly presents virtual and real mixed scenes for users; the human–computer interaction connection supports real-time interaction between users and the whole system. In summary, AR system is constantly improving and optimizing with the rapid development of science and technology. In this study, the combination of virtual and real features and human–computer interaction features of AR technology is applied to children’s picture books to provide learners with rich and intuitive learning resources, promote learners” deep understanding of knowledge, and create realistic learning situations and scientific experiments to explore the environment, to promote the occurrence of learners” deep learning.

### Psychological Features Analysis

The brain function of preschool children aged 4–5 years is not fully developed and immature, which lacks logical analysis ability. However, they are rich in imagination and good at fantasy and challenge, who have active creative thinking. Having strong imagination is the most important psychological characteristics of children at this stage, which comes from children’s feedback on environmental stimuli. But due to cognitive limitations, the image thinking ability of children is weak in this period, and their perception of the world is relatively simple. Thinking is mainly divided into abstract thinking and concrete thinking. Concrete thinking ability has been existing in a person from his or her childhood, and abstract logical thinking ability is formed in a person’s school age. Besides, at this stage, children obtain certain understanding abilities, but the ability is still relatively elementary. They can generate feedback from the external environment through the observation of the surrounding environment and things. They have preliminary reading comprehension ability and this period is the best period for them to receive an education. Hence, 4- to 5-year-old preschool children can grow through reading picture books, which are helpful to their learning habits and also thinking and cognitive ability (Okumu et al., 2017; Weissman et al., 2020).

**FIGURE 5:**
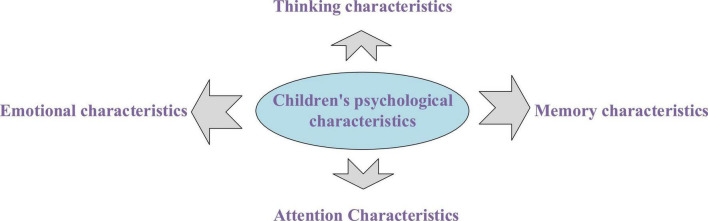
Children’s psychological characteristics.

Children’s psychological characteristics include four aspects as shown in Figure 5.

(1) Thinking Characteristics
Generally speaking, children begin to focus on concrete thinking when they are at the age of 4 years, whereas abstract logical thinking will only show up slowly after they get to 5 years old. The formation of abstract thinking is mainly manifested in the fact that children begin to have a specific concept of an object, have a correct judgment of right and wrong, and gradually form reasoning thinking. Mastering the simple classification of objects and the simple causal relationship has greatly improved the understanding ability of children compared with that of the before. The design and development of AR children’s picture books need to be paid attention to the cultivation of children’s abstract thinking (Meinawati et al., 2021).
(2) Memory Characteristics
There are two characteristics in the memory development of preschool children aged 4–5. First, unconscious memory is dominant, whereas conscious memory appears and develops continuously. The memory of these children is short, things are remembered quickly and forgotten quickly for them, and children’s memory is easy to be interfered with by external factors. Second, children’s memory is mainly based on image memory, and the logical memory of words of them is in the developing stage. In this stage of age, children’s memory lacks purpose, and they can only remember the things that they are interested in. When observing things, they can only remember the specific image of the object. At this stage, letting children read in a relaxed and happy environment can improve children’s memory ability and achieve the effect of moistening things silently (Norbury et al., 2017; Zahra et al., 2021).
(3) Attention Characteristics
The research shows that 4- to 5-year-old preschool children passively pay attention, and they are more interested in objects with bright colors and vivid appearance. Additionally, there are only 10 to 20 min of stable attention duration for their memory. Children’s attention is easily disturbed by the outside world. Therefore, parents are generally worried about children’s inattention. How to effectively attract children’s attention and enhance children’s interest in reading is an important problem to be solved in the design and development of AR children’s picture books (Farooqui, 2018).
(4) Emotional Characteristics
The mood of preschool children aged 4–5 years fluctuates quite frequently. Generally speaking, they have the following emotional characteristics: First, they often stimulate the outside world, and their mood fluctuates greatly, unlike adults, who can control their emotions. Second, children like to express their emotions with body language, which is easy to be expressed in explicit behavior. Third, children’s emotions are highly contagious (Mana et al., 2013).

With the analysis of the emotional characteristics of preschool children aged 4–5 years, their psychological characteristics can be summarized as follows: they are self-centered and have strong curiosity; their thinking can diverge quickly, their brains are active, and the development of their perception needs multisensory stimulation. Their behavior is easily affected by emotion, and their self-control ability is weak. They hope to be praised and affirmed. They are lively and actively considering the games as the main form of their activity. The design and development of AR children’s picture books based on the mobile terminal will further focus on children’s cognitive characteristics.

### Applying Augmented Reality Technology

(1) Digital Content Enhances the Narrative Function of Picture Books
Traditional paper-based children’s picture books are mainly based on picture content, supplemented by a small number of words, which provide children with a linear and passive reading experience. In essence, AR children’s picture books refer to a new display and auxiliary method, and its characteristic of the combination of reality and real-time interaction makes picture books more suitable for AR display. Compared with traditional paper picture books, the advantage of AR children’s picture books lies in the fact that through technical treatment, the story content of picture books is presented more intuitively while retaining the book fragrance of paper picture books, which is the integration of technology and art.
With the support of AR technology, the linear story content in picture books can be presented in a three-dimensional and intuitive non-linear form. Self-participation in story construction is more conducive to children readers’ understanding of the contents of picture books. Digital contents such as 3D animation, video, and audio enhance the narrative function of picture books. After scanning picture books using smart devices, the static picture will become a multimedia display with sound and video as a whole, and the story seems to appear in the real environment. The characters and scenes in the picture book are integrated with the real space where the children readers are located so that children can have an immersive experience. This brings a strong sense of immersion and substitution to reading, which also meets children’s creative imagination needs (Altintug and Debreli, 2017; Huang and Jiang, 2019).
(2) Human–Computer Interaction Enriches the Sensory Experience of Picture Books
Traditional paper children’s picture books need children to read page by page. AR children’s picture books change the single reading to multiple interactions, and their human–computer interaction brings a game-oriented and emotional interactive form. Children interact with the role of picture books through interactive behaviors such as touching and finger clicking, thus becoming the subject of interaction. Using visual and auditory senses to perceive the contents of picture books, a brand-new reading experience that is different from the traditional reading mode of picture books can be obtained for the children readers. The real-world experience creates a virtual and real interactive sensory experience, which enriches children’s reading experience. The virtual reality scene and multisensory experience satisfy children’s sense of participation. Interaction of traditional paper books with AR changes the way of reading and story presentation (Quitadamo et al., 2017; Chakraborty et al., 2018).
In the AR environment where real objects and virtual images coexist, information is put into the system, and then, the system responds to users with its presentation, which constructs close coordination between things in the physical world and images in the virtual world, thus embedding the children pictures’ books with AR characteristic. There are many methods to pass the feeling directly to the users. In addition, the most important basic principle is to integrate “user experience in real environment” with the AR environment. With technology’s growing mature, there are various methods of realizing the interaction and diverse hardcore configuration to construct a system with an AR environment. So far, there are four main methods to realize interaction. Touch screen interaction – nowadays, capacitive screen is becoming very popular in mobile devices, which also provides the basis for AR interactive technology, and touch screen interaction has become the mainstream form of such devices. At present, the mature ways include double-click feedback, light touch, and multitouch. Multitouch technology can complete the interaction between the system and many contact points at the same time. Users can put both hands into operation and even can meet the needs of multiusers. Eye-tracking interaction – there are two major methods to realize eye-tracking interaction technology. One is to capture the user’s eye focus position. The other is to measure the trajectory of the eye with the head as a reference. Therefore, the core of eye movement is the configuration of user attention, focusing on specific content. This technology can collect and record the eye movement during the user’s using process and then sort out the user’s attention retention time and observe points facing different contents. This technology provides users with a picture of the integration of AR and the real world, which is more immersive. However, due to the complexity of this technology, it is not large-scale commercialized. Hand gesture recognition interaction – gesture recognition interaction controls the device by sensing the user’s gestures, which is highly acceptable to users. At present, two kinds of methods have been developed to realize hand gesture recognition. One of the methods is using special gloves to complete movement tracking and collection, but this also increases the complexity and costs of the technology; second, special algorithms are used to collect the user’s hand movements. The algorithms can identify the direction or action of fingertips and palms for interaction. Speech recognition interaction – the device receives the user’s speech in the form of the voice signal and processes it as the corresponding command or text output, which is a kind of speech recognition interactive technology. Three major characteristics need to be paid attention to: first is model training technology, second is the model matching criterion, and third is feature extraction technology.
(3) Multimedia Integration Improves the Information Transmission Efficiency of Picture Books
Traditional paper picture books mainly focus on static illustrations in content, with relatively simple forms of expression, and some too abstract contents are not conducive to children’s understanding. With the integration of AR technology, the picture book becomes a three-dimensional animation work combining sound and picture, the content and information of paper picture book increases, and the addition of multimedia makes the characters in the story three-dimensional. The presentation of the scene also has a sense of substitution. Multimedia integration has strengthened the expressive ability of picture book stories, effectively improved the transmission efficiency of picture book information, and made it easy for children readers to understand. This multimedia integrated reading form can make picture book reading that appears in various ways, give children various sensory stimuli, and stimulate children’s multidimensional perception. It can cultivate children’s imagination, perception, and creativity and contribute to children’s thinking ability in images (Oshi, 2018).

### Interview and Questionnaire Design

To understand the present situation of children’s reading demand, parents’ cognition of AR children’s picture books, and their expectation of AR children’s picture books’ functional demand, a questionnaire survey is conducted on the parents of children from a kindergarten in Shaanxi Province, with a total of 120 parents whose children are aged 4–5 years. There is no significant difference in children’s learning ability and knowledge level, and the education level of parents is the same. A total of 120 questionnaires are distributed, and 112 valid questionnaires are finally recovered. The questionnaire is suitable for collecting survey data from most target groups (Chen et al., 2018; Yu et al., 2018). There are two forms of the questionnaire: paper questionnaire and online questionnaire. A professional questionnaire is used in this survey.

## Results and Discussion

### Results of Questionnaire Survey

**FIGURE 6:**
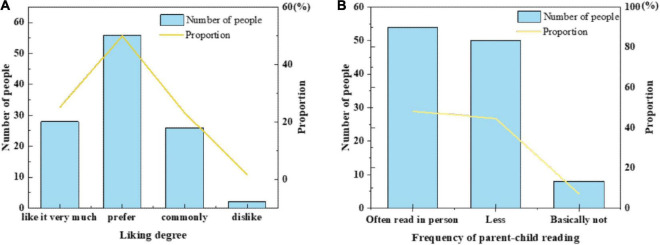
A survey of children’s and parents’ attitudes toward reading. **(A)** Preschool children’s preference for reading picture books. **(B)** Parents’ attitude toward parent–child reading.

This survey collected 112 valid questionnaires. After sorting out the 112 valid questionnaires, the research results are as follows:

(1) Figure 6 displays the survey results of children’s preference for picture book reading and parents’ attitudes toward parent–child reading.

**FIGURE 7:**
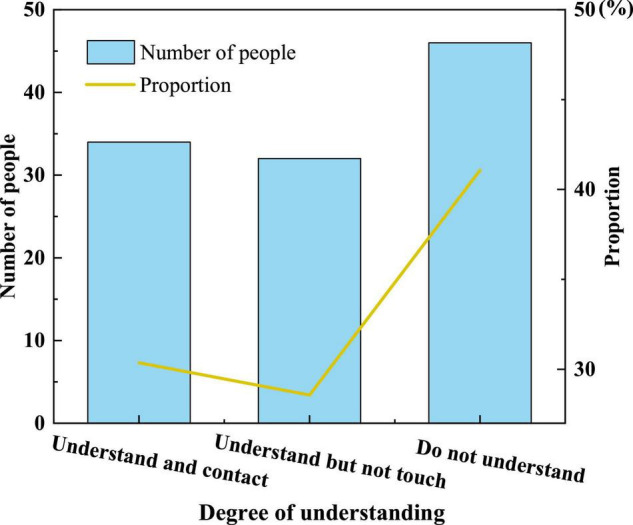
Parents’ understanding of AR technology.

Figure 6 implies that more preschool children prefer picture book reading, and only a few do not like picture book reading. Parents are also willing to participate in parent–child reading, but the times may vary due to working hours, so the market prospect of picture books is good.
(2) Figure 7 denotes the survey results of parents’ cognition of AR technology.

**FIGURE 8:**
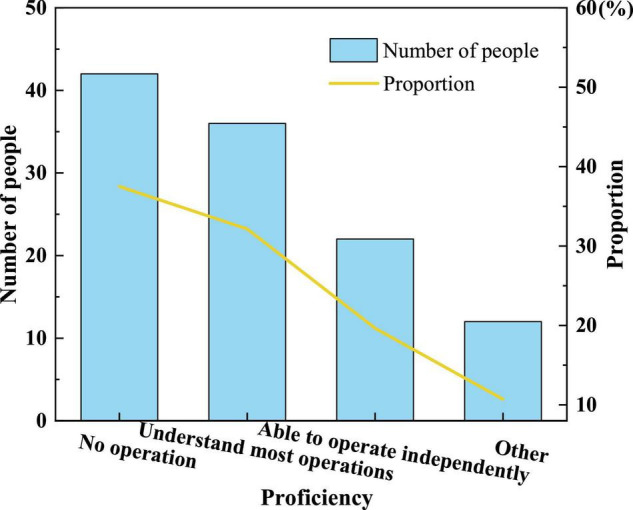
Preschool children’s proficiency in operating mobile devices independently.

Figure 7 signifies that some parents only have a general understanding of AR technology and have not been exposed to it so much, and even fewer parents have purchased AR picture books for their children. It shows that the market share of AR children’s picture books is not high, and other factors affect parents’ purchases.
(3) Figure 8 reveals children’s proficiency in using mobile devices independently.

**FIGURE 9:**
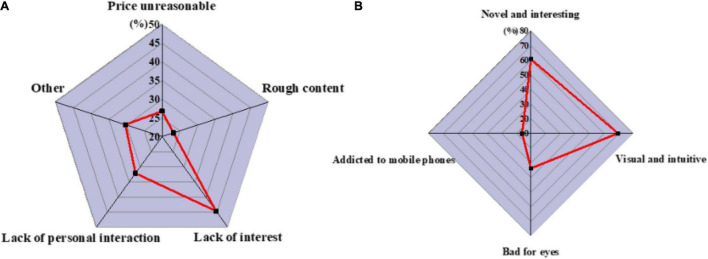
Parents’ views on AR children’s picture books. **(A)** Problems which people think exist in AR picture books on the market at present. **(B)** AR children’s picture books bring to their children.

Figure 8 illustrates that the number of people who know most of the operations is almost the same as those who cannot operate, which shows that in the design and development process of AR children’s picture book, both AR interactive content interaction and APP interaction need to pay attention to the operation ability of children users on mobile devices, which cannot be generalized.
(4) Figure 9 shows the factors that parents buy AR children’s picture books and the research results that parents think AR children’s picture books can bring to their children.

**FIGURE 10:**
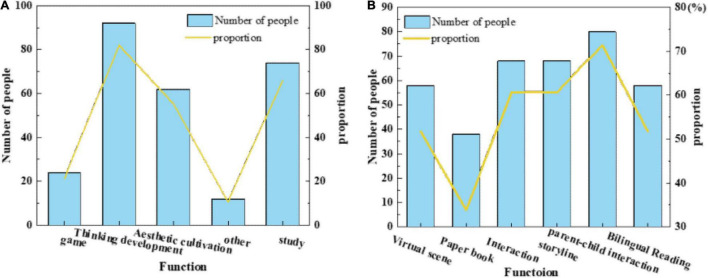
Parents’ expectations of AR children’s picture books. **(A)** The functions that parents want AR children’s picture books to have. **(B)** Parents think that AR children’s picture books need to pay attention to the design and development direction.

Figure 9 presents that many factors restrict parents from purchasing AR children’s picture books, such as lack of interesting interaction, lack of parent–child interaction, and rough production of virtual content. Besides, parents also believe that AR children’s picture books can bring benefits to children’s reading. However, there are still concerns about the influence of eyesight and addiction to playing mobile phones, which needs to be highly valued by design developers.
(5) Figure 10 displays what functions that parents expect AR children’s picture book products to have and what aspects of design and development should be paid attention to.

Figure 10 reveals that parents generally expect AR children’s picture book products to have the function of developing children’s thinking ability and making children fond of the learning. Besides, aesthetic training functions are also the design priorities, whereas the expectation for the game function is generally low. Additionally, parent–child interaction is a design aspect that parents attach great importance to. These are the factors that need to be considered in the design and development of AR children’s picture books.

According to the questionnaire survey, results clearly show that parents and children have great demand for picture books. Most parents have a positive attitude toward AR children’s picture books based on mobile terminals and think that excellent AR children’s picture books can entertain children and bring novel experiences to children. It is the parents who make the final purchasing decision. At present, many factors restrict parents from purchasing AR children’s picture books. Hence, the products should make parents to buy and use with peace of mind based on making children interested. Nowadays, most parents are busy at work, who spend relatively limited time with their children, so they pay great attention to the parent–child interaction of AR children’s picture books. It is hoped that the product design will pay more attention to the development of children’s thinking, aesthetic function, and the cultivation of their learning ability.

### Design Strategies and Principles

**FIGURE 11:**
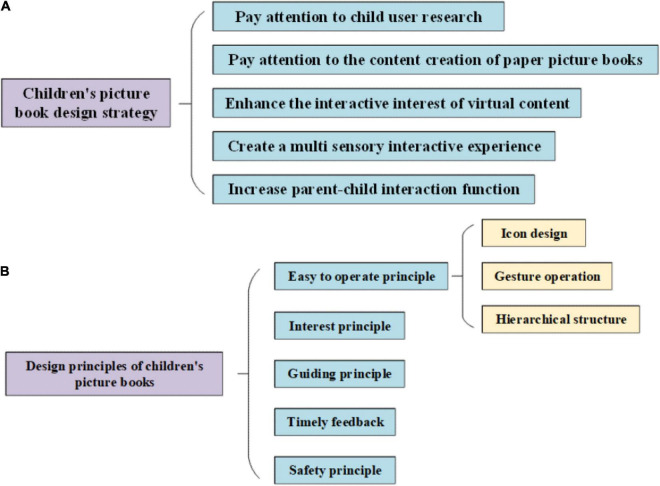
Design method of AR picture book based on the mobile terminal. **(A)** Children’s picture book design strategy. **(B)** Interactive design principles of children’s picture books.

The design and development of AR children’s picture book based on mobile terminal involve interdisciplinary and multifield technologies. Only by reasonably combining AR technology with interactive technology on the mobile terminal, can the artistic features and educational significance of AR children’s picture book be brought into full play with high efficiency and high quality. As for the problems existing in the design of AR children’s picture books at this stage and combined with the design characteristics of excellent cases, this work puts forward the following design strategies and principles suitable for mobile AR children’ s picture books, as shown in Figure 11.

The design and development of AR children’s picture books based on mobile terminals involve interdisciplinary and multifield technologies. Only by combining AR technology with mobile terminal interaction technology, can AR children’s picture books play their artistic characteristics and educational significance efficiently and qualitatively. Given the problems existing in the design of AR children’s picture books at this stage and combined with the design characteristics of excellent cases, five suitable design strategies are put forward for AR children’s picture books on mobile terminals, which are explained in detail:

(1) Attention should be highly paid to the children users.
User research is an indispensable part of any product design process, so it is the design and development of mobile AR children’s picture books. From the initial design concept of the product to the functional architecture, to the completion and production, user research should be carried out through the entire development process. Because designers and developers are not children users, whose lifestyles and way of thinking are far from adult users. Hence, it is difficult to resonate with target users without in-depth research. According to Piaget’s theory of children’s cognitive development, children’s cognitive development stages can be divided into four parts according to their variety of ages. Children in different stages have great differences, and their psychological and physiological cognition are similar and different. If user research is not made properly and thoroughly, products likely designed will make children feel uncomfortable or difficult to attract their attention and stimulate their interest in reading and using. According to the cognitive development of children users, the corresponding design is particularly important. To better distinguish the needs of children users and provide them with high-quality picture books and age-appropriate APP products, it is necessary to master children’s reading psychology and their habits in operating APP in the early stages of product design development and customize differentiated products (Jeffri and Rambli, 2017; Yang and Wang, 2017; Waters et al., 2019).
(2) Content creation of AR children’s picture books should be strengthened.
The production of AR children’s picture books based on mobile terminals can not only use AR technology to show off skills but also ignore the creation of picture book content. It is necessary to combine high-quality picture book content with AR technology. Paper picture book is the carrier of AR virtual content presentation and superposition, and the interaction of virtual content needs to be realized by a paper picture book. Fun AR interactive forms need to be set off by the integration of pictures, text, and virtual content in paper books. In the creation of AR children’s picture books, attracting children’s interest is the starting point, and the content must be the core, and the educational function of picture books should be highlighted in content. The organic combination of the readability of picture book content and the interest in pictures is the key to improve the quality of original picture books. The design of picture book content should reserve space for the deduction of virtual content. In the early creation of picture books, we can consider in advance what plots can interact through AR (Sabrina, 2017).
(3) The interactive entertainment of virtual content should be promoted.
Through the recognition function of the camera embedded with mobile equipment, AR virtual content can be presented on the screen of the mobile device. Various forms of virtual content can improve children’s interest in learning, including different presentation dimensions, different presentation styles, and rich dynamic images such as video and audio. AR virtual content design focuses on the need to ensure that the paper picture book story content plot integrity under the premise of appropriate design and add for the virtual content interactive plot, to ensure its interest and perfect. Virtual content presented by AR is essentially an extension of paper picture book content. In the design and development of AR interactive function, the first thing to be considered is the correlation between virtual content and picture book content, and it is necessary to ensure that the AR display form and interactive effect are consistent with the paper picture book content. The second is how to use AR technology and multimedia forms to strengthen the visual effect of picture books to improve the interactivity and entertainment of paper picture books. Technology serves content. The presentation of virtual content should be reasonable and meet the personalized needs of children’s reading groups. The effective combination of static picture book content and AR interactive effect can attract the reading interest of children readers (Li and Zheng, 2020).
(4) Building multisensory interactive experience.
In the stage of children exploring the world and enlightening thinking, sensory experience is the most basic and direct way for children to feel the world. AR children’s picture book products can improve children’s reading experience by guiding children’s visual, auditory, and other senses. Through this reading form, children users can not only intuitively feel the story content of picture books, but also stimulate children’s interest in exploration and thirst for knowledge, and truly cultivate children’s creative thinking. Therefore, the design of AR children’s picture books focuses on the presentation of multisensory interactive expression. On the one hand, it is necessary to properly connect and transition paper content and virtual content; on the other hand, it emphasizes the design of artistic expression, interactive design, three-dimensional production, sound feedback, and so on, to realize the multisensory experience function of picture books and create a perfect reading experience. For preschool children, they need to use icon symbols that are easy to understand visually. In color matching, they can consider the use of colors with high purity and brightness. In the hearing, they can add sound interactive prompts and feedback and integrate them into real and natural sounds. In a tactile sense, they can try to complete the design of paper picture books with different materials, to stimulate children’s multiple sensory experiences.
In recent years, as parents pay increasingly more attention to the education model, the educational concept of edutainment is becoming gradually more popular. While paying attention to children’s physical and mental development, designers need to reposition the interaction in the AR environments according to their psychological and behavioral characteristics for children of different ages. How to create effective and interesting interaction and create a multisensory interactive experience will be the focus of AR children’s picture book design (Lubrica et al., 2017).
(5) The performance to realize the interaction between parents and children should be improved.
In parent–child reading communication, parents are the key to determine the reading effect. Therefore, the difference in reading behavior is determined by parents’ attitude to AR children’s picture books. Through the questionnaire survey on parents, it is found that parents generally hope to be able to add a parent–child interaction module in AR picture book application, through the use of APP can meet the needs of parent–child reading. Although some AR children’s picture books enhance children’s reading experience by simulating their parents’ voices, this kind of bypass effect cannot completely replace their parents’ reading status. Because in the traditional parent–child reading communication mode, parents will timely and appropriately expand stories in the picture books, comparing the story with life experience and the truth to cultivate children’s habits. Some interactive functions in AR children’s picture book application are less helpful to children’s oral expression ability. Therefore, when designing the virtual content of picture books, it is necessary to enrich the parent–child interaction form of application and increase some parent–child communication functions. If the real sound acquisition function is added, the picture book story is assigned by the child user or the parent user. The system scores the performance of the child and the parents according to the collected audio content, and the collected audio can be saved for children and parents to listen to repeatedly. Furthermore, the multiplayer game can be added into the design, where children and parents can play different roles in picture books, thus making interesting interaction in the form of role-play according to the picture book (Ginn et al., 2017; Kildare and Middlemiss, 2017).

The interactive design principles of AR children’s picture books based on mobile terminals mainly include five points, which will be analyzed in detail.

(1) Easy to operate the principle
Children have limited knowledge of the world, and the simpler things they operate in their thoughts, the easier it is to get their love. Children’s picture books should stand in the angle that children like and change the operation mode of the APP interface into the minimalist mode that the children like. Because of limited cognition, children usually do not like things that are too complicated or particularly rational. When adding interface functions and visual elements, it should be fully considered whether these meet the needs of children users, eliminate irrelevant functions and elements, and reduce the thinking burden of children users. Adding complicated functions for the sake of visual effects will distract children’s attention and affect the user experience. Consideration should be made from three aspects: icon design, gesture operation, and layer and architecture how to design AR children’s picture book APP to meet children’s needs interactively and how to operate it simply.
(2) Interest principle
Children’s self-restraint ability is weak, and children aged 4–5 can concentrate for only about 5–20 min. The older they are, the longer they can concentrate. The survey shows that most AR children’s picture book products, except paper picture books, are rarely used many times after being used once. Therefore, improving the interest in interface interaction and AR interaction is one of the keys to keep children users, which can make children pay more attention and prolong the use time of products. For preschool children aged 4–5, the interestingness of interface interaction can be reflected in some small details, such as interesting dynamic effects when clicking function icons and jumping to pages. The role-playing mode can be added to AR interaction, and role-playing is a kind of game-like interaction mode. Children can choose their favorite virtual characters in picture books, and in the virtual scene constructed according to the contents of picture books, they can participate in game checkpoints to promote the development of storylines, to complete the reading task.
(3) Guiding principle
For AR children’s picture book products, to enable children users to quickly use the matching APP and help them quickly enter the product experience, it is necessary to guide children who use the APP for the first time on the functional interface. Effective guidance can keep children users and increase their interest in using the APP. On the one hand, children’s attention time is short, and the success of guidance directly determines children’s interest in using

• the APP. On the other hand, designers should also pay attention to guiding methods and learn to use simple methods to improve children’s interests. Children’s ability to recognize characters is limited, so it is required not to do too much character guidance when guiding, and proper integration of some animation or music effects can improve the guiding efficiency.
(4) Timely feedback
Feedback refers to an appropriate response of the APP interface to the user’s operation steps when the user performs human–computer interaction. The interface of APP needs to provide feedback for every operation step, which is a very important part of the user experience. The purpose of feedback is to let users know that their actions have produced results. Timely feedback can inform the children whether the operation is correct and guide them to the next step, to improve the fluency of using APP while avoiding excessive feedback.
(5) Safety principle
Preschool children’s self-control and judgment ability are weak. Long-term use of electronic products will lead to children’s users indulging in them, and there are potential safety hazards and addiction risks, which will affect their eyesight and physical and mental health. The safety of electronic products is particularly important. When designing an AR children’s picture book APP, designers should fully consider product safety. Inappropriate circumstances such as pornography and violence should not appear in the content. It is also necessary to control the time when children use products and set up the function of parents’ intervention. After the children use the product for more than the set time, the screen will be locked, and there will be prompts such as the need to rest and relax their eyes.

## Conclusion

With the development of AR technology and its maturity in the use of mobile devices, combining AR technology with paper picture books, characters, scenes, and other contents in picture books can be presented in three-dimensional form and on the screen of mobile devices, and readers can interact with virtual contents in a real environment. The application of AR technology keeps the advantages of traditional picture books, but has the advantage of the reading process changing from static to dynamic, and making reading more interesting. However, due to the technical limitations and cost problems, the combination of AR technology and children’s picture books is mostly tested in the market at present, and few products are well received. Focusing on children is the key to the design and development of AR children’s picture books. AR children’s picture books make the picture book content, virtual content, APP visual design, and interactive design of AR children’s picture book based on mobile terminal meet the needs of target children users, and at the same time, it is more interesting, instead of just using technology as a marketing gimmick, which is worthy of in-depth exploration in the field of book publishing, design, and development. Primarily, the related concepts and theories are expounded for the research contents. The preschool children aged 4–5 are selected for exploration, and the psychological characteristics of the target children users are analyzed in depth. Then, the existing cases of AR children’s picture books are studied, and the existing problems in the design of AR children’s picture books are put forward based on the questionnaire survey results. According to the needs of children users, the design strategy and interaction design principle of AR children’s picture book on the mobile terminal are constructed, which is more in line with children’s audience positioning.

## Data Availability Statement

The raw data supporting the conclusions of this article will be made available by the authors, without undue reservation.

## Ethics Statement

The studies involving human participants were reviewed and approved by Beijing Polytechnic Ethics Committee. The patients/participants provided their written informed consent to participate in this study. Written informed consent was obtained from the individual(s) for the publication of any potentially identifiable images or data included in this article.

## Author Contributions

The author confirms being the sole contributor of this work and has approved it for publication.

## Conflict of Interest

The author declares that the research was conducted in the absence of any commercial or financial relationships that could be construed as a potential conflict of interest.

## Publisher’s Note

All claims expressed in this article are solely those of the authors and do not necessarily represent those of their affiliated organizations, or those of the publisher, the editors and the reviewers. Any product that may be evaluated in this article, or claim that may be made by its manufacturer, is not guaranteed or endorsed by the publisher.
